# Cutaneous Reactions to COVID-19 Vaccines in a Monocentric Study: A Case Series

**DOI:** 10.3390/jcm11133811

**Published:** 2022-06-30

**Authors:** Carmen Cantisani, Camilla Chello, Teresa Grieco, Luca Ambrosio, Norbert Kiss, Antonella Tammaro, Giulio Tosti, Giovanni Paolino, Giovanni Pellacani

**Affiliations:** 1 Department of Dermatology, Policlinico Umberto I Hospital, Sapienza University, Viale del Policlinico 155, 00161 Rome, Italy; c.cantisani@policlinicoumberto1.it (C.C.); camilla.chello@uniroma1.it (C.C.); luca.ambrosio@uniroma1.it (L.A.); giovanni.pellacani@uniroma1.it (G.P.); 2 Department of Dermatology, Venereology and Dermato-Oncology, Semmelweis University, 1085 Budapest, Hungary; norbert.f.kiss@gmail.com; 3 Department of Neuroscience Mental Health and Sense Organs, Sapienza University, 00189 Rome, Italy; antonella.tammaro@uniroma1.it; 4 Melanoma and Soft Tissue Sarcoma Division, Istituti di Ricovero e Cura a Carattere Scientifico (IRCCS), European Institute of Oncology, 20141 Milan, Italy; giulio.tosti@ieo.it; 5 Dermatology Unit, San Raffaele IRCCS, 20132 Milan, Italy; paolino.giovanni@hsr.it; 6 Department of Dermatology, Vita-Salute San Raffaele University, 20132 Milan, Italy

**Keywords:** COVID-19 vaccination, delayed inflammatory reaction, erythema multiforme, mRNA, parapsoriasis, purpura, sweet syndrome, vaccine reaction

## Abstract

After coronavirus disease 2019 (COVID-19) caused a global pandemic, vaccines were rapidly developed to control the spread of the virus. Although they were effective in most of the cases at protecting people from becoming seriously ill and being hospitalized, they showed side effects, too. Among other adverse vaccine reactions, cutaneous eruptions following SARS-CoV-2 have been described in the literature, but they are not well-characterized yet. We described the morphology and timing of the spectrum of cutaneous reactions following most of the COVID-19 vaccines available in Italy, which were observed in outpatients referred to our non-invasive diagnostic clinic. Most of these reactions appeared after the second or third COVID-19 vaccine dose (most of them after mRNA COVID-19 vaccines). Our data support that cutaneous reactions to COVID-19 vaccination are generally self-limited; in addition, history of allergic reaction to a specific food, medicine or vaccine should not discourage vaccination in the general population, although patients with immune dysregulation should be accurately selected and monitored. Further research is necessary to better assess the true prevalence and preventive measures of skin reactions to COVID-19 vaccination.

## 1. Introduction

Following the emergence of severe acute respiratory syndrome due to coronavirus-2 (SARS-CoV-2) virus that resulted in a global pandemic of coronavirus disease-2019 (COVID-19), vaccines were rapidly developed to control the spread of the virus. The various available products can be classified into the following groups:

1. mRNA vaccines: Pfizer-BioNTech, BNT162b2, USA, MODERNA, mRNA1273, USA;
2. Viral vector DNA vaccines: Oxford-AstraZeneca, AZD1222; Sputnik V, Gam-COVID-Vac; Johnson & Johnson, Ad26.COV2.S;
3. Protein subunit recombinant vaccines;
4. Inactivated and attenuated vaccines [1].

Pfizer BioNTech and Moderna messenger RNA (mRNA) vaccines utilized a novel technology, in which mRNA encoding the SARS-CoV-2 spike protein enveloped in lipid nanoparticles penetrates all the cell membrane into the cytosol and produces a spike protein for subsequent antigen presentation and immune system activation, widespreading all over the body [2].

Since COVID-19 vaccines were introduced to the market, several reports have documented a high number of adverse reactions, with more frequently described symptoms such as fever, myalgia, headache and recurrence of chronic inflammatory diseases. Indeed, COVID-19 vaccines are able to induce a strong inflammatory reaction with the release of Th1 cytokines, which are typically over-expressed in certain pathologies such as in psoriasis, vitiligo and lichen planus [1]. A careful analysis of the reported cases of severe allergic reaction to vaccines has shown that they are very rare and mostly related to certain ingredients, such as polyethylene glycol and polysorbate, which are used as excipients [3]. The most frequently reported cutaneous manifestations that present after more than 24 h after the vaccine include the “COVID arm”, or the onset of sometimes erythematous, painful patches, located at the injection site; urticaria-like skin reactions; exanthema-like eruptions, erythema multiforme, erythematous lesions in the lower limbs, pityriasis rosea, lymphomatoid drug reactions and flare up of psoriasis [1,4,5,6]. Herein, we aim to describe cases of patients referred to our outpatient clinic with cutaneous manifestations following COVID-19 vaccination.

## 2. Case Series

Here, we report the cases of five female and eight male patients with skin manifestations following SARS-CoV-2 vaccination observed in our outpatient clinic from March 2021 to March 2022. All patients signed an informed consent form for data and image collection. Patients were subjected to skin biopsy, laboratory tests, and imaging techniques, as necessary.

## 3. Local Skin Reaction to Injection Site and Urticarial Manifestation

**Figure 1:**
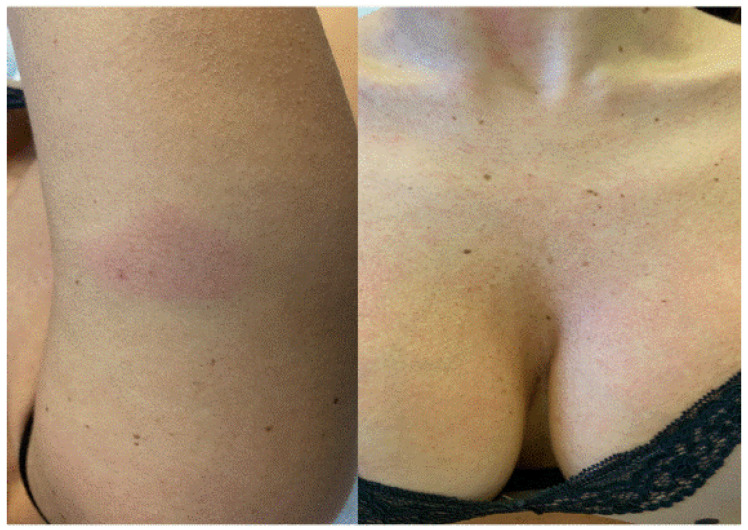
Urticarial rash: generalized itchy erythematous–edematous lesions after Pfizer BioNTech vaccine in the same patient.

A 42-year-old female patient with history of allergic rhino-conjunctivitis presented with a widespread pruritus since two days after her second vaccination with Pfizer BioNTech vaccine. Upon skin examination, the presence of an urticarial rash was appreciated over the entire skin surface (Figure 1). Blood test and ultrasound examination revealed an autoimmune thyroiditis. After four weeks of treatment with an antihistamine drug b.i.d., the patient experienced complete remission.

## 4. Acral Vasculitis and Palpable Purpura

**Figure 2:**
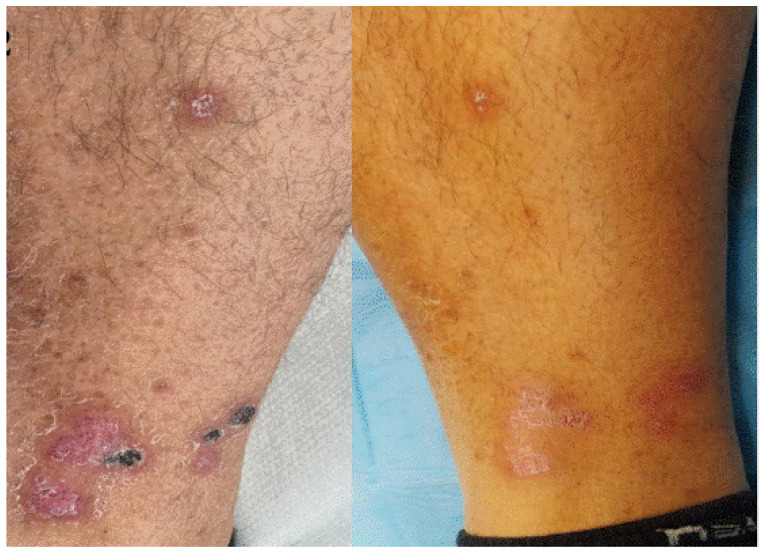
Erythematous–purpuric patches with necrotic foci in a patient with palpable purpura after Pfizer-BioNTech booster almost complete healing 1 month later.

Five patients (2F/3M) aged between 13 and 49 years old developed acral vasculitis associated with fever in the first week after booster Pfizer-BioNTech administration (Figure 2).

**Figure 3:**
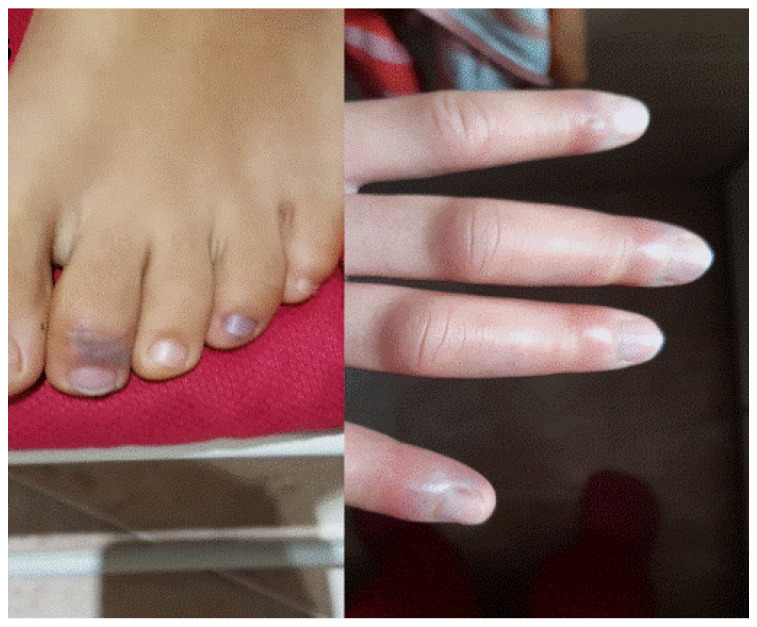
Violaceous macules on fingers and toes compatible with palpable purpura caused by cutaneous small vessels vasculitis observed 7 days after booster Pfizer-BioNTech vaccination.

Among them, especially, a 47-year-old man was referred to our outpatient clinic, as he developed dry purpuric lesions on both legs one week after Pfizer-BioNTech booster vaccination (Figure 3). Upon taking medical history, the patient mentioned that he also developed leg edema after his second dose. He had no history of any infections or new medication. Blood tests revealed increased D-Dimer test, increased erythrocytic sedimentation rate, and C-reactive protein. A diagnosis of vaccine-induced immune palpable purpura was made and confirmed by histology. Following the use of topical and systemic vasoprotective medication, his thrombocytopenia gradually recovered before making the cutaneous biopsy.

## 5. Sweet Syndrome

**Figure 4:**
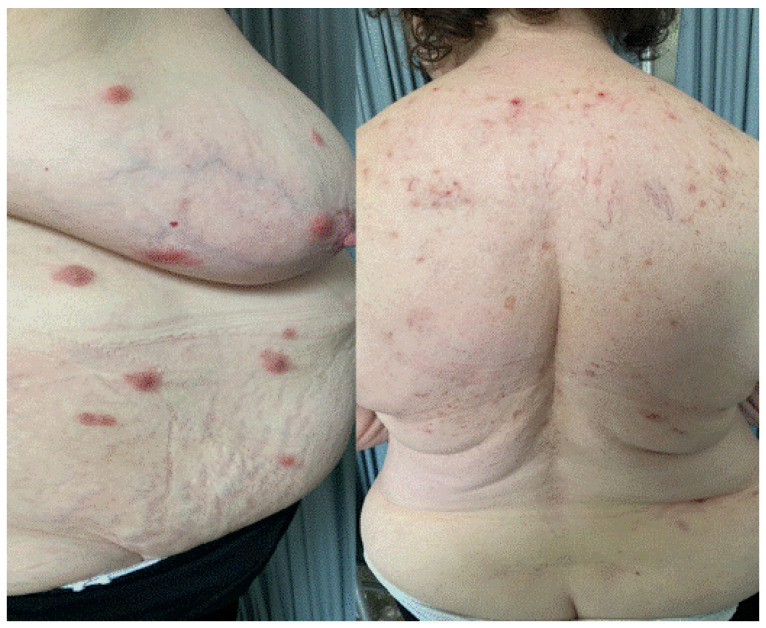
Erythematous plaques on the trunk, back and abdomen in a patient with acute neutrophilic dermatosis (Sweet’s syndrome) after Pfizer-BioNTech vaccine.

An 81-year-old female patient was referred to our clinic complaining of generalized erythematous–violaceous popular–nodular lesions. The eruption appeared two weeks after the second dose of Pfizer-BioNTech vaccine (Figure 4) without systemic involvement and fever. She suffered from severe COVID-19 six months before. A blood test revealed elevated neutrophil count (>6000 cells/μL) and elevated C-Reactive Protein. The skin biopsy showed a dense neutrophilic infiltrate with no evidence of vasculitis. Considering clinical signs and laboratory data, a diagnosis of Sweet syndrome was made. A biopsy was made to confirm the condition. Systemic corticosteroids were administered, leading to complete resolution.

## 6. Parapsoriasis

An 80-year-old woman was referred to our clinic complaining of the sudden development of generalized itching lesions two weeks after the second dose of Pfizer-BioNTech vaccination.

**Figure 5:**
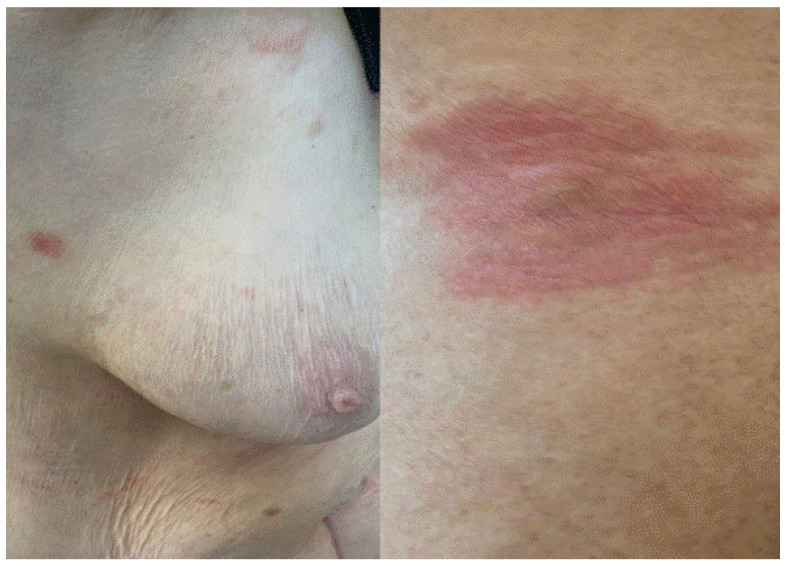
Erythematous and scaly plaque on the axillary fold histologically diagnosed as parapsoriasis.

Upon clinical examination, we detected roundish, pink to erythematous scaly plaques characterized by a fine telangiectatic network, atrophy and a few erosions (Figure 5). Blood test showed increased erythrocytic sedimentation rate and C-Reactive Protein. A skin biopsy was performed, and the result was consistent with the diagnosis of parapsoriasis. Under topical corticosteroids, a complete resolution was seen. To date, there are not yet referred cases of parapsoriasis due to COVID-19 vaccine.

## 7. Dermatomyositis-like Eruption

A 74-year-old man was referred to our clinic with a skin rash characterized by heliotrope rash and sun-exposed shawl erythema of the upper trunk. The skin manifestation developed 2 weeks after the second dose of Astrazeneca vaccine. He did not have any family or personal history of autoimmune disease. Weakness of arms and legs accompanied the skin involvement. ANA, ENA and Myositis-specific Autoantibodies were within the range. The skin eruption did not respond to any topical treatment; therefore, he asked for a second opinion. Eczema-like erythematous infiltrated plaques were also observed that appeared almost nodular on the scalp [7]. A skin biopsy was performed, showing signs of perivascular inflammation.

Considering clinical manifestation, histology and the absence of specific autoantibodies, we made the diagnosis of dermatomyositis-like eruption.

## 8. Acute Generalized Exanthematous Pustulosis (AGEP)

**Figure 6:**
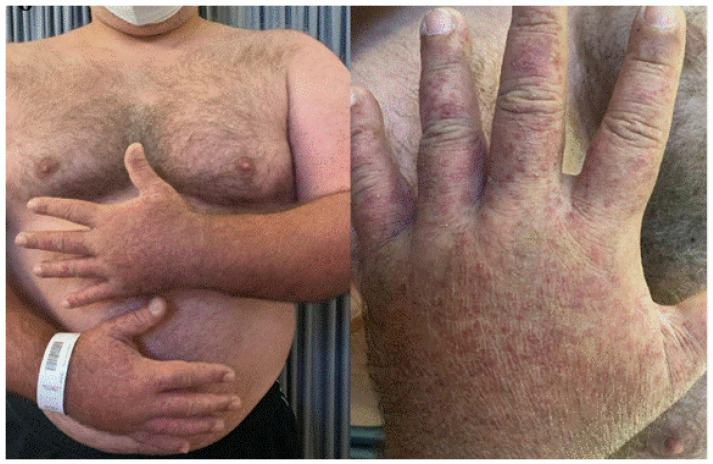
Diffuse papular–pustular lesions after second dose of Pfizer-BioNTech vaccine.

A 37-year-old man developed generalized pustulosis 1 week after second Pfizer-BioNTech vaccine administration (Figure 6). Due to high fever and rash, he was admitted to the emergency room. He presented with diffuse pustular lesions on the trunk and upper arms. His family and personal history was negative for psoriasis. Laboratory tests showed leukocytosis with neutrophilia and C-Reactive Protein augmentation. A diagnosis of acute generalized exanthematous pustulosis (AGEP) was made. Corticosteroids systemic therapy was promptly started with betamethasone 4 mg twice a day, tapering in the following days, leading to skin lesions resolution, before the planned skin biopsy could be performed.

## 9. Cutaneous Lupus Erythematosus

**Figure 7:**
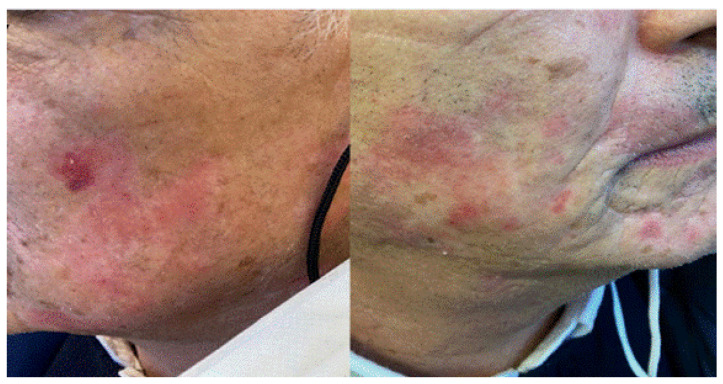
Erythematous annular patches in malar and chin areas in a patient with previous history of cutaneous lupus erythematous.

A 67-year-old man was complaining of facial skin lesions one week after Pfizer-BioNTech booster vaccination. We observed that scaly and atrophic erythematous plaques developed on both cheeks. His medical history was positive for cutaneous lupus erythematosus of the face, and therefore, he refused to undergo skin biopsy. Based on the clinical aspect, we hypothesized a reactivation of cutaneous lupus after vaccination. The 0.1% tacrolimus topical therapy resulted in an improvement of the face cutaneous lesions. (Figure 7).

## 10. Erythema Nodosum

We describe a case of 49-year-old male heart transplant who presented with tender, erythematous nodules that rapidly appeared on the distal arms and legs 2 weeks following booster SARSs-CoV-2 vaccination. The patient developed pericarditis after his second dose of vaccine. Diagnosis of classic EN presentation was made, resolving with systemic corticosteroids and conservative management. Upon a follow-up visit, only residual pigmentary lesions on the left forearm persisted.

## 11. Erythema Multiforme-like Rash

A 38-year-old male patient presented at the emergency room with fever, sensitivity to light, blurred vision, aching joints and generalized erythematous, target-like eruption one week after his second slot of BioNTech vaccine. Blood tests revealed increased neutrophils, polymerase chain reaction and D-dimer. The therapy with systemic antihistamines, corticosteroids and emollients determined a whole healing of lesions after two weeks.

## 12. Discussion

The global SARS-CoV2 pandemic has been a challenge to medical science worldwide and created new protocols to study possible therapies and vaccines. Vaccines have saved millions of lives since their introduction over 200 years; therefore, several efforts were made to speed up vaccinations for SARS-CoV-2. New technologies were developed that expanded the ability to create big numbers to be distributed worldwide. Among 117 vaccines undergoing clinical trials, the major platforms include protein subunit; RNA; and inactivated viral vector. Post authorization of mRNA vaccine, a wide spectrum of complications is continuously being reported in the literature. In this case series, we provided a brief overview on heterogeneous cutaneous findings that have been observed in our outpatient clinic since the rising number of COVID-19 vaccinations, especially after the second and third round. Most are mild to moderate and self-limiting, although severe reactions are also reported in the literature. The prevalence of vaccine reactions to COVID-19 vaccinations depends on the surveillance method, and it varies from 2.5 to 5.1 events per million; Moderna showed 2.5 whilst Pfizer-BioNTech showed 4.7 per 1 million doses [1]. After Moderna and Pfizer-BioNTech booster COVID-19 vaccines, local skin reactions and urticaria-like skin lesions may develop as reported in our cases. These reactions may reflect immediate hypersensitivity but rarely have been associated with anaphylaxis. The rate of allergic reactions to Pfizer-BioNTech vaccine is higher among patients with allergies, particularly among a subgroup with a history of high-risk allergies. The literature suggests that most patients with a history of allergic diseases and, particularly, highly allergic patients can be safely immunized at certain medical facilities and/or referral centers after completing a risk assessment questionnaire [6]. Further studies are required to define more specific risk factors predisposing to Type I hypersensitivity to COVID-19 vaccines.

Regarding vaccine-induced immune palpable purpura (Figure 2 and Figure 3), this cutaneous reaction was mainly determined by the reduced expression of the ADAMTS-13 protease. Indeed, we described the appearance of systemic manifestations following episodes of microangiopathy of the organs, including the skin, where petechial lesions may arise [8,9].

As described in the literature, SARS-CoV-2 infection can induce massive pericyte activation, vessel wall fibrosis, and multiple vascular district atherothrombosis through a cytopathic effect, inducing an exaggerated immune response with endothelial damage and prothrombotic dysregulation. It is common knowledge that SARS-CoV2 virus can cause so-called “COVID-19-associated ”coagulopathy “and ”thromboinflammation” [10].

Sweet syndrome or acute neutrophilic dermatosis, instead, is a disorder characterized by rapid onset of fever accompanied by erythematous–violaceous popular–nodular lesions, which are localized on the head and neck region and on the trunk [11]. More frequently found in women, several causative agents are recognized in its background, such as recent infections, several drugs, or neoplasms, in particular hematological disorders [11].

There are few reports of cases of Sweet following COVID-19 vaccination, including the case of a woman with erythematous lesions on the hands and feet about seven days after the first dose of AstraZeneca vaccine [9] and the one of a patient with infiltrated plaques on the body with facial sparing, which occurred about 24 h after the first dose of Pfizer-BioNTech vaccine [12].

Moreover, in the latter case, the patient had been subjected to patch and prick tests with the vaccine and polyethylene glycol, with negative results, while intradermal injection with the diluted vaccine showed a delayed type positivity [12].

The onset of Sweet syndrome after various vaccines has been described in the literature, such as that of Calmette Guerin, pneumococcal and influenza vaccines [12]. This would be concordant with the possibility that an infectious stimulus could induce, in susceptible subjects, an acute inflammatory response with systemic and cutaneous manifestations.

AGEP is a rare cutaneous eruption characterized by localized or diffuse non follicular and sterile papules. It is typically induced by drug intake, but also, infections may lead to the same cutaneous manifestation.

There are a few cases reported in the literature about AGEP after vaccine, both mRNA and viral vector vaccine. The hypothesis is that vaccination could induce the same immunologic response that is seen after SARS-CoV-2 infection; indeed, the same cytokine cascade is released in both cases [2,7,13,14]

Our case was the first reported patient with clinical manifestations of cutaneous lupus erythematosus (CLE) arising after the first dose of vaccination. In fact, it is now known that in patients with COVID-19, there is an immune hyper-activation, with increased levels of type I and III IFN, with the release of related cytokines, which in turn lead to the progression of inflammation [15,16].

Indeed, in the most severe cases of COVID-19, there is an over-expression of certain cytokines, such as IFN-gamma, TNF-alpha and IL-6, with strong pro-inflammatory action: these molecules are able to activate also the coagulative cascade with the risk of disseminated intravascular coagulation [15,16].

Moreover, risk alleles related to the IFN pathway have been associated with systemic lupus erythematosus [14]. Thus, we can hypothesize that in patients with allelic loci of susceptibility, the increased expression of IFN levels as an antiviral response may also induce an immunological activation, leading to exacerbation of previous cutaneous lupus.

Erythema multiforme is an inflammatory skin condition that usually is linked with infections, drugs, and internal diseases as well as with vaccines. In this regard, Cecilia Buján Bonino et al. reported a case of an atypical erythema multiforme related to Pfizer–BioNTech COVID-19 vaccine, highlighting how the disease secondary to COVID-19 vaccination would be an expected complication in some cases [17].

Knowledge of these reactions during mass vaccination may help healthcare professionals and reassure patients especially about different types of hypersensitivity reactions (type I and delayed type), autoimmune skin conditions, functional angiopathies, and (re) activation of viral conditions associated with COVID-19 vaccination. Reports from the American Academy of Dermatology documented more than 400 patients with skin reactions, with the Moderna vaccine responsible for most of these reactions. However, some reports highlighted the onset of cutaneous manifestations different from those commonly found, with atypical clinical pictures.

It has been demonstrated that molecular mimicry between SARS-CoV-2 spike-protein sequences and human components exists. This pathogenetic model can explain the ADRcross-reaction with COVID-19 vaccines, especially mRNA ones. Our cases well represent this possible association.

Possible limitations of our study are the small sample size and the absence of specific registers for the epidemiological assessment of adverse reactions to vaccines.

Therefore, further research is necessary to better assess the true prevalence and preventive measures for the above-discussed COVID-19 vaccine reactions, focusing the attention on spike protein enveloped in lipid nanoparticles, especially after several rounds.

## Data Availability

Data available from the corresponding author upon reasonable request.
